# Application of eco-friendly tools and eco-bio-social strategies to control dengue vectors in urban and peri-urban settings in Thailand

**DOI:** 10.1179/2047773212Y.0000000059

**Published:** 2012-12

**Authors:** Pattamaporn Kittayapong, Suporn Thongyuan, Phanthip Olanratmanee, Worawit Aumchareoun, Surachart Koyadun, Rungrith Kittayapong, Piyarat Butraporn

**Affiliations:** 1 Center of Excellence for Vectors and Vector-Borne Diseases, Faculty of Science, Mahidol University at Salaya, Nakhon Pathom, Thailand; 2 Department of Disease Control, Ministry of Public Health, Nonthaburi, Thailand; 3 Go Green Company Limited, Science Building 2, Faculty of Science, Mahidol University at Salaya, Nakhon Pathom, Thailand; 4 Department of Social and Environmental Medicine, Faculty of Tropical Medicine, Mahidol University, Bangkok, Thailand

## Abstract

**Background:**

Dengue is considered one of the most important vector-borne diseases in Thailand. Its incidence is increasing despite routine implementation of national dengue control programmes. This study, conducted during 2010, aimed to demonstrate an application of integrated, community-based, eco-bio-social strategies in combination with locally-produced eco-friendly vector control tools in the dengue control programme, emphasizing urban and peri-urban settings in eastern Thailand.

**Methodology:**

Three different community settings were selected and were randomly assigned to intervention and control clusters. Key community leaders and relevant governmental authorities were approached to participate in this intervention programme. Ecohealth volunteers were identified and trained in each study community. They were selected among active community health volunteers and were trained by public health experts to conduct vector control activities in their own communities using environmental management in combination with eco-friendly vector control tools. These trained ecohealth volunteers carried out outreach health education and vector control during household visits. Management of public spaces and public properties, especially solid waste management, was efficiently carried out by local municipalities. Significant reduction in the pupae per person index in the intervention clusters when compared to the control ones was used as a proxy to determine the impact of this programme.

**Results:**

Our community-based dengue vector control programme demonstrated a significant reduction in the pupae per person index during entomological surveys which were conducted at two-month intervals from May 2010 for the total of six months in the intervention and control clusters. The programme also raised awareness in applying eco-friendly vector control approaches and increased intersectoral and household participation in dengue control activities.

**Conclusion:**

An eco-friendly dengue vector control programme was successfully implemented in urban and peri-urban settings in Thailand, through intersectoral collaboration and practical action at household level, with a significant reduction in vector densities.

## Introduction

Dengue fever (DF) and dengue haemorrhagic fever (DHF) are considered important re-emerging arboviral diseases in tropical and sub-tropical zones and the disease is currently expanding beyond these usual boundaries (Gubler 1997; Guzman & Istúriz 2010). In Thailand, epidemic dengue was first recognized in 1958 (Nimmanitaya 1978; Kantachuvessiri 2002) and the largest outbreak of DHF occurred in 1987, when 174, 825 cases and 1,007 deaths were reported (Ungchusak & Kunasol 1988; Sucharit 1993).

Because dengue has four viral serotypes and a quadrivalent vaccine is still not available, control efforts in most countries including Thailand have focused on controlling the mosquito vectors, especially *Aedes aegypti*. From the initial programme in the 1960s, the Ministry of Public Health of Thailand has concentrated on vector control for dengue by spraying insecticide to control adults and using temephos (1% abate sand granules) to control larval stages. However, despite having established intensive vector control programmes and vector surveillance strategies all over the country, suppression of dengue transmission has not been fully achieved, as indicated by the number of reported cases in Thailand over the past ten years (more than 30,000 per year). The lack of efficacy of ultra-low-volume (ULV) and thermal fog application techniques has led to a re-evaluation of recommended strategies for prevention and control of mosquito vectors, and strategies ranging from integrated approaches to community participation have been considered (Gubler & Clark 1996). Moreover, the consequences of intensive use of insecticides have caused insecticide resistance in many insects including mosquito vectors, and insecticide residues retained in the food chain affect many life forms including soil bacteria and plants (Hemingway & Ranson 2000). For these reasons, the trend in dengue vector control has shifted away from the use of chemical-based control to biological-based control and source reduction/environmental management through community participation. In this paper, we report the successful application of an eco-bio-social or ecohealth approach to dengue prevention and control in urban and peri-urban settings in eastern Thailand.

## Materials and methods

### Study site and study design

The study site in Thailand was in Chachoengsao Province, located approximately 120 km east of Bangkok. This Province is representative of the geographic, social, economic and epidemiologic situation in most of Thailand. Dengue (DHF) incidence exhibited a strong seasonal pattern in the Province, with high transmission during the rainy season. The peak outbreaks of dengue were in 1987 and 2001, as in other provinces.

**Figure 1:**
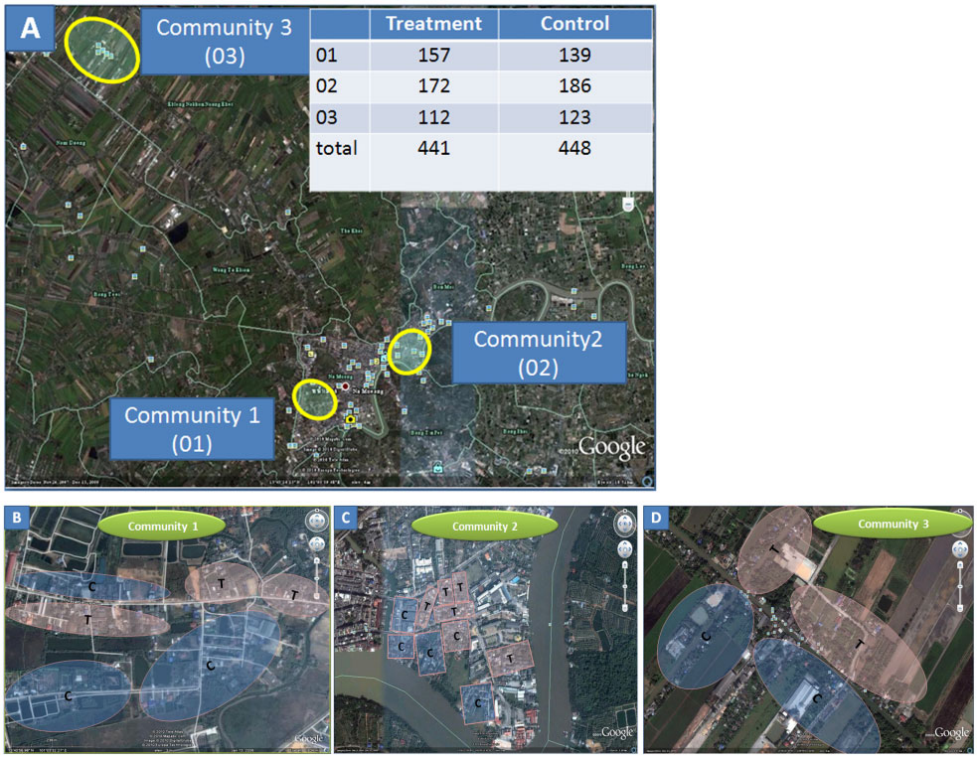
Geographic locations of urban and peri-urban communities in Muang District, Chachoengsao Province (A), showing distribution of treatment (T) and control (C) clusters in communities 1 (B), 2 (C) and 3 (D) respectively. The number of houses in each community is shown in the right hand corner of Figure 1A.

Ten intervention and ten control clusters in three communities were randomly assigned by withdrawing an equal number of clusters in each community (see Figure 1). Grid random sampling for cluster selection was not feasible in this study because vector control activities were conducted by community volunteers officially assigned according to the administrative boundaries. Community 1, Soi Li-Kae, and Community 2, Wannaying, both representing urban settings, were located in the Muang or City Municipality. Community 1 was classified as a widely distributed residential zone, and Community 2 as an unstructured densely populated area. Community 1 had moderate population density and the residential houses, mostly with garden space, were distributed randomly over a wide area. In contrast, Community 2 had high population density and unstructured houses with no garden space located next to each other. The peri-urban Community 3, Nueng Kate, was designated as an ‘ancient market’ and represented a typical community where local residents could sell their home-made products directly from their household settings. Community 3 had moderate population density. In the concentrated commercial area, houses were built as a row of wooden townhouses. However, there were houses with garden located in the adjacent non-commercial area. All three selected communities were composed of mixed residential and commercial zones.

In general, households and buildings were more tightly packed and infrastructure (connecting roads, electric service and tap water supply) was better in urban settings as compared to peri-urban areas. In all the study areas, both tap water and rainwater were used, and even though the piped water supply was reliable, people still stored water in various types of containers. An efficient municipal waste management system was in place.

In our study, sample size was calculated as proposed for cluster randomized trial (Hayes & Bennett 1999; Vanlerberghe *et al*. 2009). This intervention aimed to detect a 50% reduction in the pupae per person index (PPI), with a power of 80% and an *α* error of 0.05, assuming a coefficient of variation (standard deviation divided by the mean) of 0.25 for the clusters’ PPI. The trial was designed for six-month follow up, from May to November 2010.

### Strategies for community participation

Community participation plays a vital role in the success and effectiveness of any community-based vector control intervention. In Thailand, participation of the community is usually influenced by key people such as community leaders, local administrative authorities, municipal mayors, and local public health officers. Therefore, the first step in our community-based programme was to organize meetings with these key people to gain their participation and leadership in implementing the programme. Community mobilization meetings were held in the communities. Together with health education, these meetings intended to achieve the collaboration of government sectors and communities and to design a strategy for intervention methods according to the local ecologic and socio-demographic situation, manpower and logistic support.

In this pilot intervention programme, specific groups of ecohealth volunteers were established in each community. Through dialogue with local community leaders and coordinators, these ecohealth volunteers were identified among the health volunteers already actively involved in mobilizing dengue vector control activities in their communities. The ecohealth volunteer teams received general training on knowledge about dengue and vectors, and specific training in the use and maintenance of intervention tools, household surveillance of vector breeding habitats, and ascertaining and reporting of vector densities on specific forms. Regarding general criteria, ecohealth volunteers need to pass the training mentioned above. Importantly, they need to have positive attitude to work for their communities and to work in a team.

**Figure 2:**
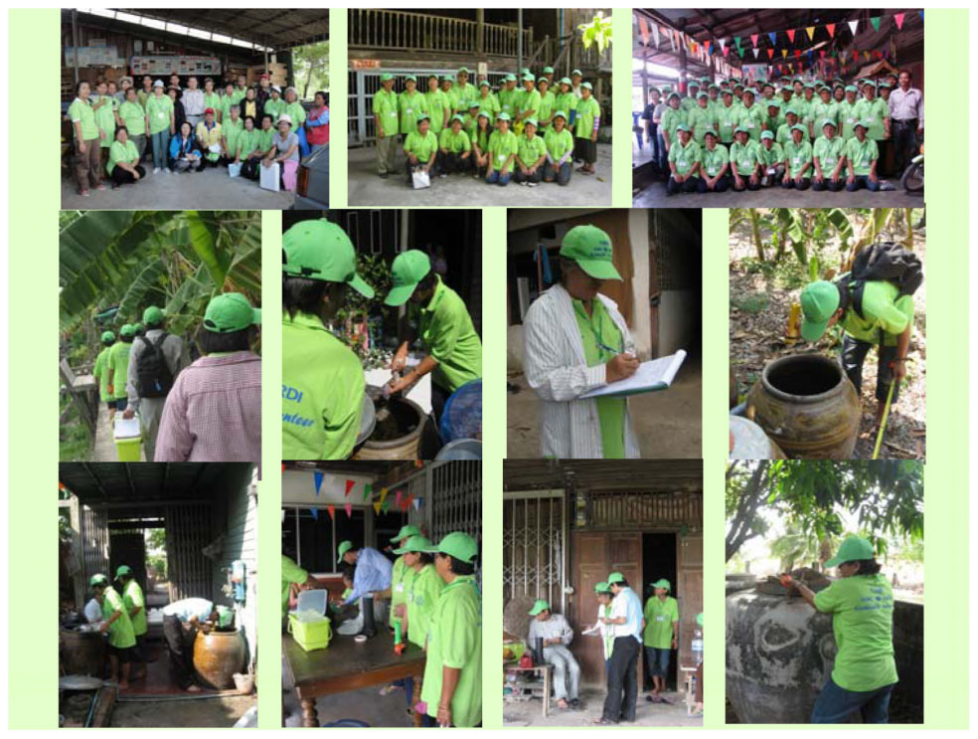
Ecohealth volunteer teams in communities 1, 2 and 3, and vector control activities undertaken in the intervention areas.

Each ecohealth volunteer was responsible for dengue vector control activities and health education in 10–15 houses surrounding their own homes. They should inform householders of general knowledge regarding dengue, vectors and prevention measures. Provision of materials and resources was supported by the public health services and local administration in collaboration with the university research teams. Health education materials and eco-friendly vector control tools were introduced into participating households located in each intervention cluster by trained ecohealth volunteers (Figure 2). During their initial household visits, the ecohealth volunteers were mentored by the research teams to perform health communication, vector surveillance and vector control using eco-friendly tools.

### Dengue vector control tools

Implementation of vector control tools and strategies was based on the choices of the local government and communities. Eco-friendly vector control tools, and the baseline data gained from the situation analysis during Phase I of the multi-country study regarding the key breeding containers (Arunachalam *et al*. 2010; Koyadun *et al.* 2012), were presented to the local government and communities. The simple and practical vector surveillance and control tools developed and produced by Go Green Co. Ltd., a spin-off company of Mahidol University, were emphasized in this intervention programme. The bio-control agent and bio-larvicide used for controlling immature stages were *Mesocyclops aspericornis* (copepods) and *Bacillus thuringiensis* var. *israelensis* toxins (Bti sacs) (Kosiyachida *et al.* 2003; Chansang *et al.* 2004) respectively. The simple vector control tools used at household level in this study were screen net covers (MosNet®) for water jars, mosquito traps (MosHouse®) and portable vacuum aspirators (MosCatch^TM^). For our previous targeted intervention, both local predacious copepods and Bti sacs were put into key breeding water containers in the treatment communities (Kittayapong *et al.* 2008). According to the entomological and household KAP (knowledge, attitudes and practices) survey conducted during Phase I (Arunachalam *et al.* 2010; Koyadun *et al.* 2012), these key containers, the containers which mostly found immature stages of dengue vectors, were categorized as cement tanks or basins, various sizes of earth jars, plastic drums or buckets. In this study, Bti sacs or copepods, depending on the choice of each household, were particularly applied into water holding containers that were used daily for general purpose, i.e., bathing, cooking, watering plants, etc., such as cement basins, earth jars, plastic drums, etc. The number of copepods and number of Bti sacs put into these containers, which followed Chansang *et al*. (2004), depended on the size or capacity of the containers. In addition, MosNet®, the screen net covers modified from Kittayapong *et al*. (1993), were introduced to prevent the development of immature *Aedes* in key breeding water jars that were mostly used for storing and drinking. Source reduction and environmental management, such as getting rid of discarded or unused water holding containers and cleaning solid waste in or around houses and neighborhoods, were carried out in the intervention clusters. Potential breeding containers were treated at one-month interval from the beginning of the intervention until the trial was completed.

### Monitoring and evaluation

Entomological surveys were conducted before the intervention and every two months after – in May, July, September and November 2010. The methodologies used for entomological survey followed Tun-Lin *et al.* (1994), Strickman and Kittayapong (2003), Chansang and Kittayapong (2007), and Barrera (2009) using the entomological survey form modified from that used in the Phase I study (Arunachalam *et al.* 2010; Koyadun *et al.* 2012). Inspectors, composed of research team members and ecohealth volunteers, worked in pairs, with one person inspecting the containers and collecting pupae, and the other recording and observing. The numbers of containers, positive containers, larvae and pupae were recorded. Each pupa was collected and reared to the adult stage in a small 50 ml tube for species identification. The number of pupae was counted and the pupae per person (PPI) index calculated.

Effectiveness of the vector control intervention was evaluated by the reduction in entomological indices, i.e. the pupae per person index. Surveys of immature stages were also conducted. Comparison between the treatment and control clusters was made according to the immature indices as well as the number of positive containers. The role and activities of ecohealth volunteers, both individual and team work, were monitored and evaluated by the university research team throughout the trial.

### Acceptance of vector control measures

A structured questionnaire was designed to collect data on acceptance of the vector control measures used in dengue vector control programmes. In total, 320 households were randomly selected from all treatment and control clusters and the interviews were conducted at the end of the intervention period. Heads of each household were preferably interviewed. The interviewer gave the respondents a free range of response to all questions; answers were coded by trained interviewers and put into major response categories. The acceptance of eco-friendly vector control tools was also evaluated by observing the feedback of householders during household visits.

### Statistical analysis

SPSS software version 14.0 was used to analyse the outcome of the community intervention. Entomological indices such as the house index (HI), Breteau index (BI), container index (CI) and mean PPI of the treatment and control clusters (ten treatment and ten control clusters) were compared cross-sectionally at two-month intervals from May to November using the independent T-test for determining impact on vector density. For the PPI, pupae collected per human population in each cluster were calculated for each cluster and then summarized into the means of intervention and control clusters. The change in all indices between treatment and control clusters during each surveyed interval was compared using the paired-T-test. The homogeneity of two sample variances was tested and a *P*-value of less than 0.05 was determined to be a significant difference. Differences between the treatment and control clusters regarding the acceptance of intervention measures were calculated by chi-square test, and a *P*-value of less than 0.05 was considered statistically significant.

## Results

### Dengue vector control intervention

During implementation, an education campaign was integrated in the intervention programme. Each household in the intervention clusters received basic education about dengue prevention and control during the periodic household visits by community ecohealth volunteers. At the same time, eco-friendly vector control tools suited to the characteristics of the household and key breeding containers identified in the clusters were offered and distributed. The local government, stakeholders and NGOs were motivated to participate in implementing the vector control intervention.

The intervention started after the first survey in May 2010. The two-month interval follow-up surveys were continued up to six months. During the intervention period, the potential breeding containers in the treatment clusters were re-treated every month. The number of follow-up households was recorded. A reduction in the number of participating households was observed. This was a result of high migration of people in the urban communities, especially the residential zone of Community I. However, the number of surveyed houses could still reach 80% coverage.

The key breeding containers focused for treatment during the intervention period were water storage jars, cement bath tanks or basins, and buckets. In the treatment clusters, MosNet®, untreated with insecticide, were applied underneath the aluminum lids, especially of standard-sized water storage jars, as these commercial lids could not prevent the development of immature dengue vectors (Strickman and Kittayapong 1993). Bti sacs and/or copepods, depending on the household choice, were mainly applied in the cement bath tanks or basins, while buckets were emptied and waste containers discarded. A significant reduction in the mean pupae per person index was found in the treated clusters when comparing the data collected at two-monthly intervals after intervention in treatment and control areas. Similar to our previous study in rural and semi-rural settings (Kittayapong *et al.* 2006, 2008), the application of screen covers for standard jars and bio-control for basins, together with source reduction and cleanup campaigns, significantly reduced the density of dengue vectors. Table 1 shows the action taken for an eco-bio-social or ecohealth approach in dengue vector control.

**Table 1:** Action for integration of eco-bio-social or ecohealth strategies in dengue vector control

Control strategies	Agents	Activities	Modes of action
1. Ecosystem management	Local government	1. Garbage and environmental management	Inter-sectoral collaboration among existing local government units responsible for activities relevant to vector breeding
		2. Provision of piped water supply	
		3. Public land space maintenance	
2. Source reduction with social mobilization	Community householders	1. Removal/reduction of non-essential water containers receptive to mosquito breeding	Household health education campaign by ecohealth volunteers
		2. Protection of water containers from presence of larvae	
3. Vector control by integrated physical and biological methods	Community ecohealth volunteers	1. Applying tight screen covers or lids (MosNet®)	1. Training and demonstration
		2. Applying bio-control agent (copepods)/bio-larvicide (Bti sacs)	2. Close supervision by experts

Public spaces, such as parks, community meeting places, religious places, etc. located in each cluster, were taken care of by the Municipality and the teams from near-by participating households in the intervention clusters. The ecohealth volunteer teams visited each household and its near-by public space three times during the intervention period and the follow-up surveys covered at least 80% of participating households.

### Outcome analysis

#### - Impact on vector density at household level

The total number of households in all three communities was 889, consisting of 441 households in the intervention clusters and 448 households in the control clusters (Figure 1, Table 2). Before intervention, 3,173 containers in the control area were inspected, yielding 583 pupae in 109 containers and leading to the mean pupae per person index of 0.38; whereas, in the treatment areas, 3,922 containers were inspected and 122 containers were positive with 648 pupae, leading to the mean pupae per person index of 0.37.

**Table 2:** Study clusters classified by dengue incidence, degree of urbanization and household characteristics in Chachoengsao Province, eastern Thailand

Community Name	Dengue incidence/urbanization/major characteristics	Number of randomized treated clusters (houses)	Number of randomized control clusters (houses)
Soi Li-Kae, Muang District	High dengue incidence/urban/moderate population density, houses with garden widely distributed over large area, mixed residential and commercial zones	3 (157)	3 (139)
Wannaying I and II, Muang District	High dengue incidence/urban/high population density, unstructured houses with no garden space located next to each other, mixed residential and commercial zones	5 (172)	5 (186)
Nueng Kate, Muang District	Low dengue incidence/peri-urban/moderate population density, mixed townhouses and houses with garden, mixed residential and commercial zones	2 (112)	2 (123)

**Figure 3:**
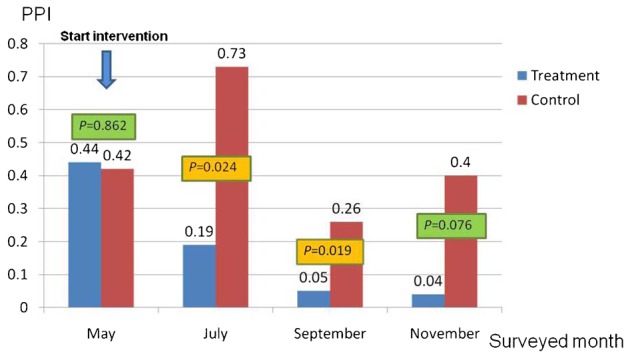
Comparison of the pupae per person index between treatment and control clusters at baseline and at two-month intervals during the intervention.

During intervention, the mean pupae per person index and all immature mosquito indices were determined at two-monthly intervals in both treatment and control areas. At the six-month follow-up, entomological indices decreased in all clusters. Larval indices, i.e. HI, BI and CI, in both treatment and control clusters were significantly lower than at baseline. There were no significant differences in HI, CI and BI indices between treatment and control clusters at each surveyed interval (Table 3). A reduction in mean pupae person index was found in the treatment area from May to November. During the peak transmission season, the mean pupae per person index was significantly different in treatment and control areas, i.e. 0.19 vs. 0.73 – *P* = 0.024, and 0.05 vs. 0.26 – *P* = 0.019, in July and September respectively (Figure 3).

**Table 3:** Control measures applied to potential breeding containers and follow-up entomological survey in the treatment (T) and control (C) clusters.

Items	Baseline		Month 2 follow-up		Month 4 follow-up		Month 6 follow-up	
	T	C	T	C	T	C	T	C
No. of inspected houses	441	448	403	400	332	368	368	335
No. of inspected containers	3,922	3,173	3,572	2,826	2,610	2,341	2,992	2,011
No. of pupa-positive containers	122	109	66	122	31	50	32	43
No. of pupae	648	583	245	970	60	346	42	361
No. of residents	1,758	1,535	1,565	1,535	1,215	1,457	1,485	1,290
House Index (HI)*	37.19	38.84	33.25	32.00	20.41	21.20	11.68	14.03
Container Index (CI)**	9.20	11.19	8.03	9.24	6.30	5.51	3.01	5.38
Breteau Index (BI)***	81.86	78.79	71.22	65.25	49.10	35.05	24.46	21.49
No. of containers applied Bti sacs	1,969		921		522		588	
No. of containers applied copepods	–		347		168		253	
No. of screen net covers applied on containers	943		–		–		–	

*At the six-month follow-up, the HI in both treatment and control clusters was significantly lower than at baseline, *P* = 0.000.

** At the six-month follow-up, the CI in both treatment and control clusters was significantly lower than at baseline, *P* = 0.002 and *P* = 0.001, respectively.

*** At the six-month follow-up, the BI in both treatment and control clusters was significantly lower than at baseline, *P* = 0.002 and *P* = 0.001, respectively.

#### - Acceptance of the intervention measures by householders

In order to assess the acceptance of the eco-friendly vector control measures used in this intervention in comparison to those used in general, a total of 320 respondents were randomly selected for interview from treatment and control clusters in three of the study communities. Of the total number of respondents, 70.3% were heads of household, and most (66.9%) were female. The mean age of respondents in the intervention area was 58.6 years, and in the control area was 53.05 years. Of the total number of respondents, 64.4% were educated at primary school level, 20.3% at high school level or higher, and 10% at secondary school level; only 5.3% were uneducated. Occupations of the respondents included business owner or trader (31.6%), employee (27.2%), government officer (6.3%), agriculturalist (1.6%), while 18.4% were unemployed.

The respondents were asked about their perception of dengue prevention and control, and all had a positive attitude towards vector control to prevent dengue. Respondents from both treatment and control clusters agreed to a survey of water-holding containers in and around their houses, and considered it necessary to discard waste containers and get rid of larvae from water holding containers. The percentage of people in the treatment clusters who agreed that it was only health volunteers who were responsible for dengue prevention in the community was significantly lower than in the control clusters (12.9% vs. 26.1%, *P* = 0.013). Similarly, a higher percentage of people in the treatment clusters compared to the control clusters (67.1% vs. 52.1%, *P* = 0.006) thought that applying copepods and Bti to water-holding containers was not complicated. However, most people agreed that covering containers with tight screen covers or lids could reduce the number of adult mosquitoes and this measure was the most preferable choice (Table 4). At the end of intervention, meetings were held with householders, government staff and researchers in all communities. It was concluded that the intervention required a longer period of time, and that long-term outcomes of the intervention were necessary, although only short-term outcomes after the intervention are reported here.

**Table 4:** Dengue vector control measures preferred by household respondents in the treatment and control clusters.

	Control (N = 165)		Treatment (N = 155)		
Control measures		%		%	*P*-value
***Measures for immature stages in water holding containers***					
Apply Abate® sand granule		98.8		95.5	0.074
Apply Bti sacs		23.0		72.9	**0.000**
Apply copepods		4.2		71.6	**0.000**
Use net covers (MosNet®) and lids		98.2		95.5	0.144*
Use insecticide-treated net covers		64.8		95.5	0.762
Change water frequently		96.4		98.1	0.283*
Get rid of un-used containers		98.8		95.5	0.073*
***Measures for adult vectors***					
Apply chemical repellents		93.3		75.5	**0.000**
Use mosquito coils		93.3		78.1	**0.000**
Use insecticide-treated curtains		52.7		47.7	**0.000**
Use mosquito traps (MosHouse®)		71.5		67.1	0.391
Use bed nets		78.8		65.2	**0.007**
Fogging		99.4		85.2	**0.000**

*Fisher’s Exact test

## Discussion

Thailand has a long history of research on dengue prevention and control. Attempts to control dengue through community participation have failed in the past (Phanthumachinda *et al.* 1986; Ungchusak & Kunasol 1988). The case fatality rate of dengue in Thailand declined from 13% in 1958 to 0.34% in 1998 (WHO 1999), an improvement resulting more from the long experience of clinical diagnosis and management than from the vector control programme. Appropriate tools to control mosquito vectors were still needed to suppress dengue vector populations below the threshold required for dengue transmission in the long term, and it was recommended that vector control policy in Thailand emphasize environmental management methods and community involvement (WHO 1999). Successful community-based vector control intervention has been demonstrated in rural settings in northeastern and eastern Thailand (Butraporn *et al.* 1999; Kittayapong *et al.* 2006, 2008); implementation of the programme in eastern Thailand emphasized integrated biological and physical control methods and community participation approaches. However, to our knowledge, there has not yet been a report on successful dengue intervention in urban and peri-urban settings in Thailand. In addition, there has not been any record of adoption of eco-bio-social or ecohealth strategies to control dengue despite an outstanding need for such an integrated approach (Spiegel *et al.* 2005). Therefore, we report here our first attempt to develop a vector control intervention suitable for urban and peri-urban communities using simple eco-friendly vector control tools and eco-bio-social or ecohealth strategies.

The results from our phase I study of the multi-country eco-bio-social approach to assess dengue transmission dynamics especially in Thailand indicated that domestic water use and storage containers such as typical water storage jars, cement baths/basins and buckets were the key breeding containers (Arunachalam *et al.* 2010; Koyadun *et al.* 2012). Adding Abate® (temephos) in potential breeding containers, and fogging to kill adult mosquito vectors, are still used as routine vector control measures in most dengue endemic areas. However, these methodologies involve using chemical substances and do not promote a healthy environment. Moreover, the cost-effectiveness of chemical control is still in question. In this intervention programme, integrated biological and physical control methods with reduced use of chemicals were successfully introduced at household level in the selected urban and peri-urban communities. Key to the success of the programme was the specific on-site training in distribution and correct application of vector control tools given to the ecohealth volunteer teams.

Observations on the vector control measures used in households were made during the study. We found that chemical control products such as insecticide sprays, mosquito repellents, and mosquito coils were used routinely, and that people in the control clusters used these products more than those in the treatment clusters. It is possible that the choice of vector control products that are non-chemical is quite limited and that the products may be more expensive. It is also possible that, as more than half the respondents preferred to use screen net covers for their water jars, suitable physical tools can be developed and applied at a household level in the future.

In our community-based programme, a combination of horizontal (bottom-up) and vertical (top-down) approaches was integrated in order to obtain immediate success. The degree of vertical and horizontal management (Gubler 1989; Gubler and Clark 1996) was adjusted according to the degree of urbanization in order to develop control strategies that were practical for urban and peri-urban settings. In general, vertical management was increased with the degree of urbanization. However, the structure and characteristics of each cluster need to be considered in order to apply suitable vector control tools and strategies.

To obtain sustainability of an integrated vector control programme, community participation and community ownership should be emphasized (Gubler *et al.* 1989). In Thailand, the first community-based control programme was not sustained, possibly due to the high degree of programme operation by the public health authorities and the lack of partnership from the targeted community (Gubler and Clark 1996). In this study, the intervention programme was implemented by local ecohealth volunteers in the communities, under active monitoring by staff of the local public health authorities and universities. Stakeholder analysis and focus group discussions indicated that community members who were normally involved in public health services and administration played an important role in driving dengue prevention and control programmes at local and provincial level. These key local persons need to be strengthened and empowered to mobilize vector control activities in their own communities.

Many studies have shown the efficacy of integrated physical and biological vector control programmes, and demonstrated that they can be applied successfully in communities. However, it is obvious that the sustainability of community-based vector control programmes does not rely solely on the vector control tools used, but on the understanding and awareness of people in the communities, which is key to the success of the programmes. Long-lasting participation of community members in all activities of the vector control programmes could reduce the incidence of dengue. Our findings demonstrated that community participation in dengue vector control in urban and peri-urban communities using eco-friendly tools could be initiated but the long-term effect will not be possible without continued support from both communities and local authorities. To obtain programme sustainability, collaboration among community sectors, i.e. local administrative authorities, public health services, communities, and external organizations e.g. academic institutions and NGOs, needs to be encouraged and evaluated over time.
